# Enacting semi-periphery: conditions of post-accession migration from Poland to the UK

**DOI:** 10.12688/openreseurope.19457.1

**Published:** 2025-02-26

**Authors:** Viktoryia Vaitovich

**Affiliations:** 1 University of Milan, Milan, Lombardy, Italy

**Keywords:** migration, EU integration, Poland, free movement, labour exploitation.

## Abstract

Since Poland’s accession to the EU in 2004, the sizeable community of Polish migrants in one of the top destination countries – the UK – have been subject to growing discrimination and hate speech in the form of negative media coverage, as well as unequal labour practices. This article explores the impact of the 2004 enlargement of the EU on the migratory experiences of Poles by examining the way the cross-EU free-movement regime contributes to their vulnerability and exploitation. It aims to uncover the way European integration explains the unequal treatment of Polish migrants in the ‘old’ Member States. The paper addresses the following questions: what factors have driven the increased number of Polish migrants in the UK in the context of the 2004 EU enlargement? Do economic discrepancies between the EU Member States, which fuel intra-EU migration, likewise shape migrant vulnerabilities? Adopting the theoretical lens of the World-Systems Analysis and the notion of biopower, this paper argues that the peripheral position of Poles in the EU single market during the early post-enlargement period has been reinforced by the migration experiences. This is an outcome of structural pressures that make the perspective of migration appear as aspirational and promising.

## Plain Language Summary

This article looks at how Poland’s entry into the EU in 2004 affected the migration motivation of Poles and their consequent experiences in the UK. It discusses the challenges they have faced, including discrimination, negative media coverage, and unfair working conditions. The study explores whether economic differences between EU countries made Polish migrants more vulnerable to exploitative practices and examines how the EU’s free movement impacts their experiences. Building on 41 interviews with Polish migrants in the UK, and through the lens of social theory, it argues that while migration is often seen as a chance for better opportunities, it has also kept Polish workers in lower-status positions within the EU job market.

## Introduction

The transformations in Poland linked to the largest EU enlargement (in terms of territory, number of countries, and population) have contributed to a significant increase in the cross-European mobility of goods, capital, services, and people. This has affected the life choices and experiences of people in the new Member States and resulted in the migration of a sizeable number of Poles to the UK in particular. This migratory movement has been explained mainly by economic factors, including less restricted access to the UK labour market, economic discrepancies between ‘old’ and ‘new’ Member States, unfavourable economic contexts in Poland, and the development of low-cost airlines and communication tools ( Beaumont *et al.*, 2017; Engbersen *et al.*, 2017).

At the same time, the increasing phenomenon of unequal labour practices in the UK affects EU citizens, among others, whereby Polish nationals represent the most common group subject to various forms of modern slavery ( Guilbert, 2019; Shankley, 2021). The demonstrated unequal position of Poles by both civil society actors and governmental agencies (which include the data on the high rates of Poles among the cases treated under the National Referral Mechanism) led to the question of the structural position of Poles within the post-2004 enlarged EU ( Chartered Institute of Building, 2018).

 Applying the framework of World-Systems Analysis (WSA), this paper argues that Polish migrants' peripheral position within the EU single market persisted even after their arrival in the "core" of the EU following enlargement. This pattern has been reinforced by their migration experiences, shaped by structural pressures that are often seen as aspirational and full of opportunity. Moreover, the argument that peripheries’ “incorporation into the capitalist economy is associated with putting a (migration) drain on them” ( de Haas, 2008) sheds light on the preconditions for post-2004 labour migration flows, notably in the case of Poland as a former country of the Communist bloc. Representing a peripheral post-communist state, Poland’s integration into the free movement framework shifted its position towards the semi-periphery within the expanding capitalist system. Hence, through this theoretical lens, the mobility of Poles can be understood as a practice which makes them “Polish migrants” as products of core-semi-periphery relations.

The empirical evidence, based on the collected contributions of 40 interview participants, allows for tracing the link between the changed policy framework introduced by the 2004 EU enlargement and the migration decision-making incentivised by the ‘self-optimisation’ in the new context. This translated into the decision to leave the country of origin and to explore better opportunities within the EU labour market. These testimonies also illustrate how these structural relations operate upon lives. The mobilities can be understood as an adaptation of life choices and strategies in response to the evolving dynamics of the international labour market. In this case, it reflects the expansion of the EU labour market, which has driven movement from economically peripheral regions to the core.

Finally, it is acknowledged that the analysis of the structural factors and economic forces may appear inadequate in understanding the individual level of the migration decision-making process, and the way the changed circumstances brought by Poland’s accession to the EU, were internalised at the individual level. Therefore, the complex migratory experiences can be analysed through the interplay between the structural forces of the free movement regime, and individual agency, motivations, and socio-cultural conditions.

## Analytical framework

The characteristics of post-2004 Polish migration have been analysed from different perspectives, particularly in terms of the push-and-pull migration model, considering the socio-economic situation in Poland and relevant discrepancies within the EU. The lack of a “coherent theory on international migration” resulted in the development of various theoretical approaches across disciplines ( Massey *et al.*, 1993). Stephen Castles argued that the multidimensional nature of contemporary migratory processes should be analysed within a broader interdisciplinary framework, while no discipline can, in a holistic manner, singularly account for a complex set of drivers, modes, and implications of migration ( Castles, 2003: 22).

Analysing the recent significant mobility of Poles, as well as their previous migration, Immanuel Wallerstein’s World-Systems Analysis appears an adequate and insightful framework ( Wallerstein, 1984). Wallerstein introduces three categories: the capitalist ‘core’, ‘semi-peripheral’, ‘peripheral’, and, finally, “isolated nations in the ‘external’ area, which were not (yet) included in the capitalist system” ( de Haas, 2008: 7).

The argument that peripheries’ “incorporation into the capitalist economy is associated with putting a (migration) drain on them” sheds light on the preconditions for post-2004 labour migration flows ( de Haas, 2008), notably in the case of Poland as a former country of the Communist bloc. Representing a peripheral communist state, Poland’s integration into the free movement framework shifted its position towards the semi-periphery within the expanding capitalist system. Thus, the above-mentioned framework appears particularly useful in analysing recent movements of Polish migrants to the UK, which are characterised by the context of the enlarged EU and the accession of several post-Communist states, that represented the countries with the sizeable emigration directed towards the “old” Member States.

In line with that premise, the relationship between the core and peripheral zones is defined as “the exchange of products containing unequal amounts of social labour” ( Wallerstein, 1984: 15), whereby the tension stemming from this unequal exchange between different zones constitutes a cornerstone of the capitalist system ( Hopkins & Wallerstein, 1982; Wallerstein, 1984). Nevertheless, the interdependency between these mentioned areas is crucial not only in understanding the course of the capitalist world-economy development — when understood in a broader sense of respective processes, the relationship between zones can also be operational in light of the differences between a ‘high-wage zone’ in the core and a ‘low-wage zone’ in the periphery ( Hopkins & Wallerstein, 1982).

I employ Wallerstein’s model of core-periphery relations to capture the logic behind phenomena such as economic disparities within the EU, high unemployment rates in Poland fuelling intra-EU labour mobility, and the instances of labour exploitation experienced by Poles in the UK. Within the modern world-system, according to Wallerstein, classes are formed as a result of “the geographical distribution of labour control and coercion that reproduces the relations of unequal exchange” ( Hopkins & Wallerstein, 1982: 91–113). According to this logic, migrants are considered as part of the international division of labour between capital- and labour-intensive zones ( Wallerstein, 1974), while Polish migrants are situated in the redistribution of surplus value from the labour-rich semi-periphery, characterised by relatively low incomes, to the capital-rich and higher income core (the UK).

Against this background, the explanatory relevance of semi-periphery (and WSA more broadly) is reflected in its capacity to account for the process of cross-border labour mobility. It sheds light on the prerequisites to the increased mobilities of Poles, explaining them as an outcome of the transformation of Poland’s economy and its integration into the common European market. WSA is useful in understanding the patterns of economic migration processes, fostered by “system forces, processes, and structures” ( Kardulias & Hall, 2006: 24), inducing migration of individuals primarily to the labour markets with “better earnings opportunities” ( Miłaszewicz *et al.*, 2015: 65). At the same time, it also speaks to occupations typically taken by mobile Poles, who are mostly involved in the low-paid sectors of the economies of the host countries. However, it is important to note the critiques of the approach related to its neglect of endogenous factors behind mobility.

Whereas WSA allows for understanding the structural forces behind the migration of Poles, which follows the core-periphery logic within the enlarged EU, it still remains unclear how the opportunities brought with Poland’s accession to the EU and the free movement area are evaluated and perceived at the individual level. To address this analytical gap, this paper suggests a novel angle on the study of intra-EU mobility, borrowing the Foucauldian notion of productive power ( Foucault, 1980) as a tool to examine the effects of European integration on the lives in the semi-peripheral states, and in Poland in particular.

Shifting from the notion of power as punitive or repressive, this concept invites the consideration of its productive effects, those that produce knowledge, and shape aspirations and desires ( Foucault, 1980: 59). Drawing on the assumption that productive power is entangled in all kinds of social relations, playing both a “conditioning and a conditioned role” ( Foucault, 1980: 142), it is relevant to explore how it can influence one’s life strategies and the perception of the available opportunities. At the broader level, it is also important to investigate how one of the strategies of productive power, namely political governmentality, is administering control over populations to address the larger special agenda ( Pinkerton, 2018; Tomei, 2016). This approach is fundamentally different from more primitive types and manifestations of power directly applied to punish or “to *take* life” ( Foucault, 1978: 138), whereas productive power projects “a positive influence on life, that endeavours to administer, optimise, and multiply it, subjecting it to precise controls and comprehensive regulations” ( Foucault, 1978: 137).

Acknowledging this idea of the governmental power that guides, manages, and makes certain life strategies desirable, it is possible to recognise a parallel with the process of the 2004 EU enlargement which allowed several post-communist states with high unemployment rates to enter the free movement area and to gain access to the labour markets of certain Member States. Those states, including the UK, were characterised by the labour shortage, particularly in the low-paid sectors of the economy. The adopted policy, which lifted the temporal restriction on the labour market access typically applied for the new Member States, can then be considered as a governmental tool to fill the labour shortage. Opening the border and the labour markets for the citizens of the newly joined countries represented the policy which evidently broadened the spectrum of the available options and encouraged the populations in the periphery of the EU to migrate to its core in search of better economic and educational opportunities.

## Methods

This research draws on a qualitative research design, which is often applied to understand “how actors construct and interpret the world surrounding them, and how these interpretations affect their actions, identities and everyday experiences” ( Barglowski, 2018: 154). The qualitative approach involves collecting data from various sources: a literature review includes journal articles and other online sources covering the period between 2004 and 2016, related to the issues of labour migration from Poland to the UK, as well as forced labour and exploitation experienced by Poles. Furthermore, following one of the fundamental principles of the inductive qualitative method, “oriented toward an exploration of the diversity of human life” ( Barglowski, 2018: 156), I have conducted semi-structured interviews with Polish labour immigrants in the UK – “the most attractive destination country for Polish migrants after May 2004” ( Kaczmarczyk & Okólski, 2008: 602). Given the aim of the research and formulated research questions, interviews are the principal sources of data in learning about the participants’ motivations and experiences related to migration, as well as their attitudes to possible unequal practices experienced while in the low-paid sectors of the UK economy.

### Sampling strategy and criteria

A non-probability snowball technique was chosen as an approach to sampling participants ( Lapan *et al.*, 2011). ‘Non-probability’ or purposive sampling means that the participants are sampled strategically, “so that those sampled are relevant to the research questions that are being posed” ( Bryman, 2012: 418). Unlike other sampling techniques, purposive sampling is based on the defined characteristics of the research participants prior to fieldwork, thus making the selection of participants dependent on the judgement of a researcher” ( Barglowski, 2018: 166). The ‘snowball’ technique is applied to reach out to a greater number of participants through already sampled ones, who might “propose other participants who have had the experience or characteristics relevant to the research” ( Bryman, 2012: 424). The formulated research questions instructed the sampling of participants, hence the primary focus on those having work experience in the low-paid sectors of the UK economy, to trace a possible correlation between migration experience and vulnerability to exploitative practices ( Anderson & Rogaly, 2005; Lalani & Metcalf, 2012; Skrivánková, 2014).

Qualitative research methodology does not provide any established guidelines regarding the size of the sample, whereas it is in the area of quantitative enquiry that the sample needs to be statistically significant to allow for respective generalisations regarding the researched population ( Barglowski, 2018: 157). Participants’ sampling was guided by the criterion of data saturation. In this context, the understanding of saturated results gathered through a sufficiently large sample implies that “further data collection would not provide relevant results to the themes, concepts, codes or theory” ( Barglowski, 2018: 158). Thereby, sampling is terminated at the point when no novel contribution is gained.

The sample comprised 41 adults from 20 to 62 years of age, who identified themselves as Polish, among whom were 9 men and 32 women. The gender imbalance that can be identified within the sample of the present study is characteristic of recent Polish migration to the UK. While Okólski and Salt show that more than a half (from 52 to 65 per cent) of post-accession Polish migration was comprised mainly of men, the late accession period was characterised by the opposite trend, with predominantly female migrants coming from Poland to the UK ( Okólski & Salt, 2014). All the participants had a migration background and work experience in the following sectors: care, cleaning/housekeeping, construction, delivery, food production, gastronomy, hospitality, manufacturing, and sales. This occupational trend is also reflected in the broader statistics related to the main economic sectors Polish migrants are involved in. Relevant data shows that, by 2011, most of the Poles in the UK were working in hospitality, manufacturing, services, education, health, transport, communication, and agriculture ( Okólski & Salt, 2014).

While interviews represent the cornerstone of primary data collection in ethnographic research ( Fedyuk & Zentai, 2018: 172), semi-structured interviewing was chosen as a technique which makes it possible to compare the individual responses on several topics ( Lapan *et al.*, 2011: 94). Following the interest in the participants' perspectives, the topics discussed covered issues such as the motivations behind migration, the employment experiences in the UK and the role of the Polish community, one’s daily working experiences, and the impact of the migration experience on one’s life and the life of the family.

### Data handling and ethics

The anonymised interview recordings were transcribed and thematically coded using QSR NVivo software ^2^ to allow for thematic content analysis. The created codes reflected the discussed topics and recurrent themes, and fell within the scope of the following larger groups: migration motivations and factors, work experiences and conditions, attitudes to exploitative practices, and social experiences. The respective coding further allowed the identification of the ways the same theme was treated among the participants, whereas the identified patterns were used to inform theoretical accounts. Transcription and coding were done during online fieldwork to identify the point of data saturation.

Regarding the ethical concerns, the research and data collection plan underwent a review process and was found in line with the ethics guidelines of Paul Valery University, Montpellier III and the University of Porto. The process of primary data collection was guided by the principles of informed consent, voluntariness, and confidentiality.

## Polish migrants as targets of power

To understand the migration experiences of Poles after the 2004 EU expansion through the lens of biopolitics, the observation by Burrell and Schweyher suggest that the UK's policy of granting free access to migrants from newly acceded countries emerged alongside a tightening of the asylum system, a shift that can be seen as “entrenching the de facto racialised contours of immigration control” ( de Noronha, 2019: 2413–30). As a result, British immigration policy has been interpreted as a biopolitical regime ( Tyler, 2010: 61–74) in which white Polish migrants held a relatively privileged position compared to other migrant groups.

The influence of biopolitics on individuals as subjects of power is evident in migration decision-making. Building on the argument that biopolitical strategies prompt individuals “to self-optimise and self-care through cost-benefit calculations in all stages of life” ( Repo, 2018: 237), it becomes clear that this “entrepreneurial image of self” ( Tomei, 2016: 1144) plays a crucial role in shaping migration choices. Consequently, while power relations in capitalist societies are understood to shape “human behaviours through orienting the agency and self-control that actors exercise over their bodies, their desires and even their fantasies” ( Tomei, 2016: 1150–51), the agency and mobility of Poles – as a practice that defines them as “Polish migrants” within core-semi-periphery biopolitical dynamic – remains an area for further exploration.

Following the premise which stresses the need to consider the multiple factors and levels influencing migration theoretically, this paper aims to include and emphasise the aspect of the subjective interpretation of the reality and the opportunities associated with migration. With this in mind, Foucault’s notions of productive power and subjectivity as its effect help understand the way the 2004 EU enlargement changed the available options and life strategies for the nationals of the so-called A8 countries, including Poland alongside Czechia, Estonia, Hungary, Latvia, Lithuania, Slovakia and Slovenia, together with Cyprus and Malta. Conceptualised as a governmental strategy, this expansion encouraged significant emigration from these countries and made it attractive and desirable, yet simultaneously and paradoxically, it resulted in subjecting many to inequality and exploitative practices in the destination countries. The testimony of a female hospitality worker in Glasgow, conveys the idea of a lack of prospects in Poland, listing several factors contributing to a decision to leave her home country.

P12: It was mostly because of the political situation that I decided to leave my country. I would just have no future there as far as I'm concerned. It’s outrageous, they are just pushing young people out of the country because there's not enough jobs for young people. From what I understand is going on in there, I cannot agree with the right-wing views and I don't agree with the politicians in power at the moment, they are very nationalistic, and they want to leave the EU as well.

The empirical evidence based on the contributions of interview participants allows for tracing the link between the changed policy framework introduced by the 2004 EU enlargement and the migration decision-making incentivised by the “self-optimisation” in the new context. This translated into the decision to leave the country of origin and to explore better opportunities within the EU labour market.

As reflected in the following testimony, in some cases, short-term migration in the post-2004 context was perceived and used as a tool to improve the financial situation, which was followed by a return to Poland: (P5) “In 2004 when Poland joined the European Union, my brother left Poland for London, he was working in a construction factory. He just wanted to pay for his mortgage in Poland. Once he did, he returned to Poland.” However, in other cases, the opened borders and the emerged opportunity to explore better paid employment were made from the position of continuous hardships and eventuated in the decision to stay in the UK:

P19: It was in 2004, or 2005 when I really tried to get a job [in Poland]. Every single job I would find, they were very low paid. I was still living with my parents at that time. But I had to find money to pay for food, and to pay bills. I didn't earn enough, so in 2004 the UK opened the border for Poles, my friend got there straight away and then I joined him. We worked all day long and in a month, I earned my yearly wage in Poland. And that was why I decided to move out of Poland. In fact, I was never planning to go abroad for a long time. But well, it has happened. Although the initial plan was to come to the UK for 3–4 months and then go back to Poland. But I'm still here.

In her analysis of the post-2004 emigration from Poland, Rabikowska looks both at the motivations for migrations and the way migration experience interplays with the identity of migrants. Looking at the post-accession context and its impact on the aspirations of Poles, she suggests the following point of view:

It can be argued that migration is mainly instigated by a picture of normality that can be achieved in the future – however vague and incoherent that picture would be among individuals – and thus it is always somehow unfulfilled and always in progress. As abstract a category as normality is, however, in Eastern Europe it evokes very concrete responses today and provokes changes in both the reality which migrants leave behind and the one in which they currently live. ( Rabikowska, 2010: 288).

The 2004 accession of Poland to the EU is often framed in the testimonies of the research participants as a landmark event that offered exit options from a dire economic situation, or represented a means to achieve the said normality, or simply opened new opportunities that appeared attractive and promising:

P21: 2004 back in Poland I was struggling because I was studying and working at the same time. None of those things were working out and I wasn't earning much so I figured out that I can do the same somewhere else for better money. Then why not try it?

The testimonies presented above demonstrate how biopolitics shapes individual choices. This migration can be understood as an adaptation of life strategies in response to the evolving international labour market – specifically, the expansion of the EU single market, which has driven movement from the economic periphery to the centre. Viewing migration through this lens helps clarify the factors influencing the decision to leave one’s home country, often at the risk of exposure to exploitation and challenging living conditions.

Walia’s argument that legal and social status insecurity contributes to hierarchical stratification ( Walia, 2013: 9), is reaffirmed by the way Poles negotiate their status and navigate the hierarchies. In this regard, the intersection of race, gender, and religion as a power structure has been employed to analyse the process of negotiating the position of Polish migrants in the UK in the post-accession context, arguing that whiteness, cultural proximity and male privilege defined their positionality within the labour market and social relations ( Fiałkowska, 2019: 11). Thus, previous qualitative research portrays the way Polish migrants appeal to these categories “to show belonging to the same community of values and resist their migration-related lower status” ( Fiałkowska, 2019: 12–13).

It is worth mentioning that the assumed privileged position of Poles within the racial hierarchy particularly at the workplace, was narrated by one of the participants: (P37) “I am white just like Scots, but I think Asians, they have a worse experience than I do. And for those from Africa, it is very hard for them to find a job because of the perceptions of the locals. I know it's ridiculous but slowly it's changing.”

Wallerstein and Hopkins – an American historical sociologist and Wallerstein’s co-author, develop Marx’s ideas by presenting class as a transnational actor within the world economy, where the transnational division of labour is one of its core elements ( Hopkins & Wallerstein, 1982: 11). According to this logic, classes, as “categories of participation in production” ( Hopkins & Wallerstein, 1982: 83), create a complex of relations within a stratified system of core, periphery and semi-periphery. Thus, the issue of transnational exploitation of labour appears central to the functioning of the world economy, whereas within this hierarchy the system is most exploitative of the periphery ( Wallerstein, 1984: 9).

Seeing exploitation as embedded in labour exchange, however, neglects the agency of workers and makes it harder to analyse the reasons for “tolerating some degree of mistreatment” ( Davies, 2018: 306) and accepting exploitative conditions. Within the literature on labour exploitation, migration is included on the list of factors contributing to the vulnerability to various forms of exploitation ( Clark, 2013; Schwarz *et al.*, 2018), while the most common economic sectors for exploitative practices are construction, agriculture and food, hospitality, cleaning, and care-giving ( Anderson & Rogaly, 2005; Clark, 2013). Moreover, Craig *et al.* argued that migration reinforces the vulnerability to the extreme forms of exploitation and slavery, irrespectively of migrants’ legal status ( Craig *et al.*, 2007), whereas elements of modern slavery include “severe economic exploitation; the absence of a framework of human rights; and control of one person over another by the prospect or reality of violence” ( Craig *et al.*, 2007).

Most of the literature in the field of labour exploitation focuses on its severe forms, drawing on the international legal standards reviewed above, and respective national legislations, which revolve around the issues of prosecution and criminal justice. Yet, this approach has been criticised for disregarding the “routine and banal” ( Davies, 2018: 294) everyday exploitative practices. The argument of persisting ‘trivial’ practices has been reaffirmed by several participants in their description of working conditions at such workplaces as construction, food production, gastronomy and hospitality, in some cases representing violations of basic health and safety standards. According to a 30-year-old female participant, who used to work in hospitality, cleaning, and care, her experiences in all these fields were characterised by some form of everyday exploitative practices. As she notes:

P16: It was hard at the restaurant as well, because it did all depend on how many people were coming to the restaurant. It wasn't that you had some set time when you could go for a break. You could only eat or drink whenever there was a time. Sometimes you would work for hours without a break. That was quite normal there. I always tried to make sure that I had something to eat and drink because I would probably collapse. But there was no place to sit, there were just some chairs outside for us next to the bins at the back of the building; it was quite smelly as well with flies. You would not want to eat there. So, we didn't have anywhere to sit. That was quite hard because you had to get used to eating and drinking while standing or kneeling, leaning against something. And that lasted for a year. Plus, there was a broken dishwasher and the floor was always flooded. So, you always had to make sure you had good shoes because otherwise, your shoes would get soaked. […] I thought it would be more organised, but it was very chaotic, and it was your problem to survive there.

While the above statement refers to a number of substandard working conditions, the absence of regular breaks represents the aspect that goes beyond the description of the ‘trivial’ practices listed in the description, pointing to an example of more severe violation, which nevertheless has been mentioned rather often during the data collection process. Similarly, another female participant working in food production referred to the experiences characterised by the long working hours performed in substandard working conditions and with physically demanding duties:

P17: I never worked physically before, I was very young. It was very cold and a long shift to work, sometimes 10, 12 hours. And it was very hard for me to work with meat, sometimes I had to carry 10 kilos. And then another 10-kilo meatloaf and another, basically it was a hard job.

In another statement from the same sector, a 39-year-old male Polish migrant in Liverpool mentioned the conditions of freezing temperatures as a daily environment, for which the employer neglected to supply the required protection:

P19: There were many issues there (at the factory), with the protective clothing for instance. People had to enter the freezer room sometimes, but you needed a proper jacket because it's minus 27 degrees there, […] so the company should provide everyone working there with proper trousers etc. But it wasn't the case. So, the safety issue was very big there, people had to wait for weeks for their clothes. I have never seen that before.

The examples of violations mentioned in the study show that they are indeed practised and have been endured by many of those working in various low-paid sectors in the UK. Whereas these specific cases might not fall within the scope of the stricter legal definitions of exploitation or forced labour, these testimonies put on display the widespread character of the conditions described above, which present the practices which can be located between the extremes of the spectrum of forced labour and decent job, chosen and accepted voluntarily.

To sum up, according to Veneziani and Yoshihara, exploitation as an unequal exchange of labour is characterised by “systematic differences between the amount of labour that individuals ‘give’ to the economy, in some relevant sense, and the amount of labour that they ‘receive’” ( Veneziani & Yoshihara, 2017), whereas the focus on ownership in social inequality theory shows that “the capitalist class owns the means of production and is thus able to exploit wage-earning workers” ( Hamann & Bertels, 2018: 396).

Applying the above-mentioned theoretical framework, the migration of Poles can be perceived not only as an example of the flow of the labour force from the semi-periphery to the centre of the economic system, where the Foucauldian approach helps to understand this process as a subjectification of Polish migrants, that reproduces their semi-peripheral position upon arrival in the host country. The notions of biopower and biopolitics unveil how the power structure shapes an individual’s decision-making, furthering migration trajectories and lived experiences. The demand for immigrant labour in the UK, rooted in the fact that “poorly educated natives resist taking low-paying jobs at the bottom of the occupational hierarchy” ( Massey *et al.*, 1993: 447), paved the way for the development of the non-restrictive policy of access to the labour market for the nationals of the newly joined Member States, resulting in a precarious position of Polish migrants in the UK.

## Conclusion

Focusing on the effects of European integration focusing on the free mobility regime and the example of the Polish labour migration to the UK during the post-accession period, this paper explored the factors impacting the increased number of Polish migrants, zooming in on the impact of economic discrepancies between the Member States and the role this asymmetry plays in complex migration motivations.

Adopting the lens of World-Systems Analysis sheds light on the structural forces, such as the unemployment rate and low wages, behind Polish migrant mobilities which follow a movement from a labour-intensive semi-periphery to a capital-intensive core. WSA also explained the positioning of Poles in the sectors of the UK labour market. Thus, I have shown that a hierarchical system comprising the core, periphery, and semi-periphery reproduces population inequalities.

Yet, the analysis of the structural factors and economic forces appeared inadequate in understanding the individual level of the migration decision-making process, or the way the changed circumstances brought by Poland’s accession to the EU were internalised at the individual level. In line with the structural account of WSA, economic forces “define the flow of migration” ( de Haas, 2008: 7), while “individuals do *not* have a free choice, because they are fundamentally constrained by structural forces” ( Castles *et al.*, 2014: 32). Therefore, structural accounts question the role of agency, suggesting that “people are forced to move because traditional economic structures have been undermined as a result of their incorporation into the global political economic system” ( de Haas, 2008: 7–8).

This theoretical assumption was qualified by the contributions of the research participants, which showed the interplay between the structural forces of the free movement regime, and the individual motivations that can be linked, among others, to the family situation or the attitudes to the socio-cultural context in Poland. The notion of productive power coined by Foucault unveiled the need to examine more closely the choices people might be compelled to make, based on the reality produced by the power relations to which they are subjected. The collected testimonies and reflections of the research participants in some cases reiterate the inevitability of the migration. A 40-year-old female participant who used to work in production in England, sharing the process of her migration decision-making in the early post-accession period, added that now she would have made the same decision. She elaborates as follows: (P31) “If I were to make the decision now if I were in Poland right now, I would leave anyway. But now, for political reasons, because of what is going on in Poland right now.” Thus, the hierarchy of motivations could have shifted, with the political one taking the leading place in light of the current course of the government and the overall political situation in the country. However, with Poland’s accession to the EU, it follows that the spectrum of available opportunities or escape options has broadened, creating a new context that presents migration as a promising path to explore.

At the same time, the significant emotional effect of the Brexit campaign on the migrant population and its impact on everyday life should be acknowledged, particularly with regard to its effect of generating uncertainty around the migrants’ plans for the future ( Anderson *et al.*, 2019; Anderson & Wilson, 2018; Burrell & Schweyher, 2019). In addition to generally constraining migration strategies ( McGhee *et al.*, 2017: 2109–30), Polish migrants in the UK have noted an increase in personally experienced racism in the period following the Brexit campaign and referendum ( Rzepnikowska, 2018). Existing research on the emotional impact of Brexit has also revealed that it constituted “a rupture to the continuity of EU citizens’ everyday lives” ( Botterill & Hancock, 2019: 3) and fostered the construction of the figure of a ‘ *European other*’, intensifying the feeling of unbelonging among Poles ( Giralt, 2020: 42), and presenting another important aspect for analysis of migration trajectories in this unprecedented context.

## Ethics and consent

The research and its data collection and management plan underwent a review process and were found in line with the ethics guidelines of the two degree-awarding institutions – Paul Valery University, Montpellier III (18 January 2021, approval number 2021-01 CER UPVM) and the University of Porto (11 December 2020, approval number 02 /CEFLUP/21.12.2020). Informed consent was obtained from all participants, using written consent forms.

Regarding the ethical concerns, the research and data collection plan underwent a review process and was found in line with the ethics guidelines of Paul Valery University, Montpellier III and the University of Porto. The process of primary data collection was guided by the principles of informed consent, voluntariness, and confidentiality.

## Data Availability

The data collected and analysed in the framework of this study cannot be shared under any circumstances due to strict ethical and confidentiality considerations. The research involved in-depth qualitative interviews with Polish post-2004 migrants in the UK, focusing on their migration motivations and experiences in low-paid sectors. Given the sensitivity of the subject matter, including discussions of exploitative conditions and potentially undocumented work, any disclosure of primary data – even in anonymised form – poses a significant risk of re-identification and potential harm to participants. This study was conducted in strict compliance with GDPR regulations and ethical guidelines approved by the Ethics Committees of the University of Porto and Paul Valery University, Montpellier III. Ethical approval mandated that all data remain confidential and securely stored on a password-protected university server, with unpublished personal data to be destroyed at the project’s conclusion. Participants were assured that their responses would not be made public to prevent legal, social, or economic repercussions. Therefore, in accordance with these ethical commitments, the dataset cannot be made available for secondary analysis or public access.
