# Supplementary material for: Enacting semi-periphery: conditions of post-accession migration from Poland to the UK
Source: Open Res Eur. 2025 Jun 25;5:56. Originally published 2025 Feb 26. [Version 2] doi: 10.12688/openreseurope.19457.2 (PMC12255893; doi:10.12688/openreseurope.19457.2)
Supplement: Peer review history

# Reviewer report: Reviewer response for version 2

**DOI:** 10.21956/openreseurope.22416.r56026

**Published:** 2025-07-12

**Authors:** Kate Golebiowska

**Affiliations:** 1 Charles Darwin University, Darwin, Australia

While the Introduction, Methods and Analysis are now largely improved, the Conclusion section remains a mixed bag. It retains the new topic about Brexit (I recommended removing it ) and does not cross-reference biopolitics - now presented as a complementary concept to interpret the topic. I think cross-referencing to it would enhance the flow and rigour of the paper. There are also instances where the clarity of expression/intent could be improved One such example is the opening sentence  in the "Polish migrants as targets of power". Overall (and with edits that the two other reviewers have made), I am happy to support the Indexing of this current version as final.

If applicable, is the statistical analysis and its interpretation appropriate?

Not applicable

Is the study design appropriate and is the work technically sound?

Partly

Is the work clearly and accurately presented and does it engage with the current literature?

Partly

Are the conclusions drawn adequately supported by the results?

Partly

Are sufficient details of methods and analysis provided to allow replication by others?

Partly

Are all the source data and materials underlying the results available?

Yes

Reviewer Expertise:

migration, mobility, migrant settlement, labour market outcomes of migrants

I confirm that I have read this submission and believe that I have an appropriate level of expertise to confirm that it is of an acceptable scientific standard.

# Reviewer report: Reviewer response for version 1

**DOI:** 10.21956/openreseurope.21053.r51872

**Published:** 2025-04-07

**Authors:** Kate Golebiowska

**Affiliations:** 1 Charles Darwin University, Darwin, Australia

Thank you for the opportunity to review the manuscript “Enacting Semi-Periphery: Conditions of Post-Accession Migration from Poland to the UK.” The paper considers the impact of Poland’s accession to the EU on Polish migration to the UK in the context of the EU’s free movement regime. Using the World-Systems Analysis and the concepts of biopower and biopolitics, the Author proposes that structural pressures have relegated Polish migrants to a peripheral position within the EU single market. Below, I am recommending revisions to improve the structure of the paper and to better integrate and use theory and evidence to contribute to the debates on immigration, power, and the EU labour market. I commend the Author for their efforts to draw connections between the macro structures and the experiences of Polish migrants in the UK.

**Introduction.** The manuscript opens with general statements such as “The transformations in Poland linked to the largest EU enlargement have contributed to a significant increase in the cross-European mobility of goods, capital, services and people” and “This has (…) resulted in the migration of a sizeable number of Poles to the UK in particular.” Numerical evidence to substantiate these statements is needed.  Replacing general terms like “significant increase” with figures comparing pre- and post-accession migration flows will ground these assertions.

 The second paragraph offers a general statement that “(…) the increasing phenomenon of unequal labour practices in the UK affects EU citizens, among others, whereby Polish nationals represent the most common group subject to various forms of modern slavery.” This claim requires empirical support. The Author may use official statistics or other authoritative data to support it.

 The Introduction shifts between the EU labour market and the UK, though the paper’s title suggests a focus on the UK. The Author may address this by rewriting the Introduction and showing that the EU labour market opportunities and conditions serve as a broad background for considering the case of Poles in the UK’s labour market. Additionally, this section will benefit from:

 -Clarifying what is the period covered by “post-accession” migration;

 -Expressly recognising that Polish migrants were present in the UK prior to accession;

**Analytical framework.** While the WSA framework is reasonably well laid out in its classical form, characteristics of the core, semi-peripheral and peripheral states in the context of the EU labour market are missing. When incorporated and supported with concrete examples and references, the analytical framework will gain more academic rigour. For example, on p. 3. the Author mentions ‘high unemployment rates in Poland’ that fuelled their intra-EU mobility for employment, but the reader is left wondering what these rates were and when, and why the scale of vacancies in the UK was a motivating factor to move. What was the unemployment rate in the UK in the period under review and in the industries where Poles found employment? Another point relates to the notion of productive power. If it is intended as an analytical framework, it should be included in the abstract.

**Sampling strategy and criteria.** This section will benefit from further elaboration. Please explain the rationale for choosing the specific industries of employment of the participants. If hospitality and agriculture are cited as the most common sectors for Polish migrants, supporting this claim with labour market data will strengthen its validity. Additionally, clarifying when the interviews were conducted (year) will help readers understand the participants’ length of stay in the UK.

**Polish migrant as targets of power.** This section blurs the boundaries between literature review, analytical framework and results and is difficult to follow. Concepts like ‘biopower’ and ‘biopolitics’ are introduced here but belong in the analytical framework. I recommend reorganising the paper so that all concepts are introduced and defined in the ‘Analytical framework’ section. This will improve the clarity and logical flow of the paper.

 The Author introduces Marx’s concept of ‘class’ (p. 7) here, further confusing the reader. I am not convinced that the Author is well-versed in the Marxian concept of ‘class’; it is hardly explained in one passage on p. 7 and referenced on p.8. If the Author chooses to retain it, a fuller explanation and integration with the WSA framework (especially Wallerstein’s categories) is needed. Otherwise, the concept can be omitted, and efforts should be directed toward developing a more focused, in-depth analysis (pp. 7-8).

 The closing paragraph on. p.8 is a moment where concepts and data should intersect meaningfully, but the interpretation is superficial and underdeveloped. For example, biopower and biopolitics are briefly mentioned there but not sufficiently applied to the empirical material. Expanding this section to show how these frameworks help explain participants’ experiences will enhance analytical depth. The citation of Massey et al. (1993) can be enriched by drawing on Okólski and Salt (2014), which is already cited elsewhere and would offer more contextual nuance.

**Conclusion:** This section is again confusing. It includes elements that should be moved to the data section, such as new examples (see P31) and the accompanying considerations. The last paragraph introduces a new topic: the Brexit-related emotional effects, which, in my view, fall outside the paper’s scope and can removed. This will create space to develop a more profound analysis using the conceptual framework.

 The Conclusion will benefit from a style review, too. The opening sentence is too long (it makes up the entire opening paragraph), and the word “focusing” is repeated in the first line. The second paragraph needs specific examples and a more detailed discussion earlier to make sense. Specifically, the claim about how the core-periphery system produces “population inequalities” requires demonstration earlier in the analysis.

 I hope the Author receives this feedback in good faith and considers the suggested revisions to develop a sharper paper. Best wishes.

If applicable, is the statistical analysis and its interpretation appropriate?

Not applicable

Is the study design appropriate and is the work technically sound?

Partly

Is the work clearly and accurately presented and does it engage with the current literature?

Partly

Are the conclusions drawn adequately supported by the results?

Partly

Are sufficient details of methods and analysis provided to allow replication by others?

Partly

Are all the source data and materials underlying the results available?

Yes

Reviewer Expertise:

migration, mobility, migrant settlement, labour market outcomes of migrants

I confirm that I have read this submission and believe that I have an appropriate level of expertise to confirm that it is of an acceptable scientific standard, however I have significant reservations, as outlined above.

# Reviewer report: Reviewer response for version 1

**DOI:** 10.21956/openreseurope.21053.r51664

**Published:** 2025-04-02

**Authors:** Tomasz Komornicki

**Affiliations:** 1 Polish Academy of Sciences, Warsaw, Poland

The article has a very broad focus of the research. It is not entirely clear what was the main purpose of the interviews conducted. This affects their correct evaluation. The theses posed go beyond the capabilities of the methodology used. At the outset we learn that this goal was to uncover the way European integration explains the unequal treatment of Polish migrants in the ‘old’ Member States. Later in the text, more far-reaching theses and references to concepts appear. Poles are qualified as the most common group subject to various forms of modern slavery. The article does not provide evidence for such a thesis. On the contrary, some statements from interviews, as well as quotes from the literature, further indicate that the situation of Polish migrants is better than those from the global south. None of the quoted statements indicate that Poles as a group were treated particularly badly. Rather, they point to generally poor working conditions in certain sectors in the UK. Therefore, some of the theses seem over-interpreted.

 The article also fails to mention some important contexts related to emigration from Poland to the UK. These include non-economic factors that determine the decision to stay in the UK for longer than the initially planned few months. Often mentioned here is the quality of public services, higher than in Poland. Another important element is the fact that not all migrants registered in 2004 were actual migrants at the time. Some had already been in the UK before, and Poland's accession to the EU only enabled them to legalize their stay and work. 

 In the article, emigration from Poland to the UK is treated in isolation from general migration flows from Poland. Against this backdrop, the UK gained (thanks to the immediate opening of the labor market in 2004) relatively better-educated migrants from Poland (relative to, for example, Germany, where migration took place illegally and included groups that were poorly qualified and more likely to be mistreated by employers). The cited data regarding sectors employing Poles (after Okólski) does not justify the selection of individuals for the interviews conducted. These are general sectors with both bad and better-paying jobs.

 The exposition of the interview referring to the political situation in Poland is unclear. First of all, this is the situation before 2023 (right-wing rule), which should be highlighted somehow. Secondly, the singular example should be contrasted with the thousands of returns to Poland from the UK that followed Brexit. 

 Explaining the intensity of migration from Poland to the UK by the development of low-cost airlines seems wrong. Rather, airlines have responded to the demand from these migrations. Bus transportation between Poland and the UK also played a key role in their first phase.

If applicable, is the statistical analysis and its interpretation appropriate?

Not applicable

Is the study design appropriate and is the work technically sound?

Yes

Is the work clearly and accurately presented and does it engage with the current literature?

Partly

Are the conclusions drawn adequately supported by the results?

Partly

Are sufficient details of methods and analysis provided to allow replication by others?

Partly

Are all the source data and materials underlying the results available?

Yes

Reviewer Expertise:

Quantitative analysis of migration, modal structure of international transport, qualitative evaluation of migration based on surveys in Poland

I confirm that I have read this submission and believe that I have an appropriate level of expertise to confirm that it is of an acceptable scientific standard, however I have significant reservations, as outlined above.

# Reviewer report: Reviewer response for version 1

**DOI:** 10.21956/openreseurope.21053.r51871

**Published:** 2025-03-25

**Authors:** Shaddin Almasri

**Affiliations:** 1 Danube University Krems, Krems an der Donau, Austria

This article titled, ‘Enacting semi-periphery: conditions of post-accession migration from Poland to the UK’ explores Polish migration to the UK through the lens of world systems theory. It posits that the enlargement of the EU has not translated to inclusion of periphery states in the ‘core’, rather, migration patterns re-enforce Poland’s status as a periphery state. The article is clear, well-written and well researched and looks to be an excellent contribution to academic literature on inequality and migration.

 Starting from the analytical framework: This article would benefit from expansion on its guiding theoretical framework. While the premise is indeed convincing on its own, in this paper, it would require further elaboration. The EU core and periphery are not well defined and established and a number of generalizations are made throughout. For instance, the author mentions unequal exchange of goods in passing but does not specifically cite statistics. As this is a novel application of this lens, I recommend further data to convince readers of the strength and relevance of this lens in the intra-EU context, as opposed to its usual application at the global level.

 Moving on to the case study and discussion: The relevance of bio-politics and biopower remains unclear. Inclusion of this as an area of further research in the case study/discussion section of this paper is somewhat confusing to the reader and may be best placed in a conclusion. It is unclear how this relates to the World Systems Analysis discussed in the previous section and requires further elaboration if this were to be kept.

 Beyond the initial discussion, there is very little actually depicting how the immigration of Poles to the UK has re-enforced Poland as a periphery state. The data depicts poor working conditions and exploitation of migrant workers, however the link to the thesis is not clear. There appear to be many implied links and connections made, however it does not yet sufficiently prove the thesis. Furthermore, the conclusion introduces new data that would be best placed in the discussion, if it is indeed a part of the argument.

 On to the conclusion: The conclusion explicitly states that WSA explains the positioning of Poles in the labor market through a look at inequalities. However the ‘red thread’ is not yet clear – there are several lines of thinking (WSA, bio-politics, migrant decision making, un-belonging) but they are not sufficiently connected. I understand that this likely because the interview content was rich and it indeed appears to be! However the thesis is made somewhat disjointed and I’d therefore recommend that the author focuses more clearly on one or two of these elements.

 Some small notes:

- On the sampling strategy: I note that all migrants interviewed for the purpose of this study are workers mainly in the service sectors, with the exception of construction. Was this deliberate and if so, why? While not a majority, there are Polish migrants across many sectors in the UK with different levels of qualifications. Indeed, this impacts experience and I find that the paper would benefit from clarifying why there’s a particular focus on those in particular sectors.
- Overall: The paper would benefit from a tighter edit.

Overall this is article is very promising! The data collected appears to be rich and informative and may produce several papers as a result. I look forward to its final iteration and congratulate the author for this contribution.

If applicable, is the statistical analysis and its interpretation appropriate?

Not applicable

Is the study design appropriate and is the work technically sound?

Yes

Is the work clearly and accurately presented and does it engage with the current literature?

Partly

Are the conclusions drawn adequately supported by the results?

Partly

Are sufficient details of methods and analysis provided to allow replication by others?

Yes

Are all the source data and materials underlying the results available?

No source data required

Reviewer Expertise:

Migration, refuge, inequality and aid politics in the MENA region. The article may benefit from a review by someone with expertise in application of Marxian frameworks to migration.

I confirm that I have read this submission and believe that I have an appropriate level of expertise to confirm that it is of an acceptable scientific standard, however I have significant reservations, as outlined above.
